# Genome Analysis and Characterisation of the Exopolysaccharide Produced by *Bifidobacterium longum* subsp. *longum* 35624^™^

**DOI:** 10.1371/journal.pone.0162983

**Published:** 2016-09-22

**Authors:** Friedrich Altmann, Paul Kosma, Amy O’Callaghan, Sinead Leahy, Francesca Bottacini, Evelyn Molloy, Stephan Plattner, Elisa Schiavi, Marita Gleinser, David Groeger, Ray Grant, Noelia Rodriguez Perez, Selena Healy, Elisabeth Svehla, Markus Windwarder, Andreas Hofinger, Mary O’Connell Motherway, Cezmi A. Akdis, Jun Xu, Jennifer Roper, Douwe van Sinderen, Liam O’Mahony

**Affiliations:** 1 University of Natural Resources and Life Sciences, Vienna, Austria; 2 APC Microbiome Institute and School of Microbiology, University College Cork, Cork, Ireland; 3 Alimentary Health, Cork, Ireland; 4 Swiss Institute of Allergy and Asthma Research (SIAF), University of Zürich, Davos, Switzerland; 5 Alimentary Health Pharma Davos, Davos, Switzerland; 6 Procter & Gamble, Cincinnati, United States of America; Purdue University, UNITED STATES

## Abstract

The *Bifibobacterium longum* subsp. *longum* **35624**^™^ strain (formerly named *Bifidobacterium longum* subsp. *infantis*) is a well described probiotic with clinical efficacy in Irritable Bowel Syndrome clinical trials and induces immunoregulatory effects in mice and in humans. This paper presents (a) the genome sequence of the organism allowing the assignment to its correct subspeciation *longum*; (b) a comparative genome assessment with other *B*. *longum* strains and (c) the molecular structure of the **35624** exopolysaccharide (EPS_624_). Comparative genome analysis of the **35624** strain with other *B*. *longum* strains determined that the sub-speciation of the strain is *longum* and revealed the presence of a **35624**-specific gene cluster, predicted to encode the biosynthetic machinery for EPS_624_. Following isolation and acid treatment of the EPS, its chemical structure was determined using gas and liquid chromatography for sugar constituent and linkage analysis, electrospray and matrix assisted laser desorption ionization mass spectrometry for sequencing and NMR. The EPS consists of a branched hexasaccharide repeating unit containing two galactose and two glucose moieties, galacturonic acid and the unusual sugar 6-deoxy-L-talose. These data demonstrate that the *B*. *longum* **35624** strain has specific genetic features, one of which leads to the generation of a characteristic exopolysaccharide.

## Introduction

The gut microbiome contributes to host health by multiple mechanisms, including digestion, competitive exclusion of pathogens, degradation of mucins, enhancement of epithelial cell differentiation and promotion of mucosa-associated lymphoid tissue proliferation [1, 2]. Furthermore, accumulating evidence suggests that the composition and metabolic activity of the gut microbiome has profound effects on proinflammatory activity and the induction of immune tolerance within mucosal tissue [3–6]. Certain microbes induce regulatory responses, while others induce effector responses, resulting in the case of healthy individuals in a balanced homeostatic immunological state, which protects against infection and controls aberrant, tissue-damaging inflammatory responses [7].

The *Bifidobacterium longum* subsp. *longum* **35624**^™^ strain (formerly named *B*. *longum* subsp. *infantis*) is known to induce tolerogenic responses within the gut and reduces disease symptoms in irritable bowel syndrome patients [8–10]. Induction of T regulatory cells by *B*. *longum* **35624** strain (**35624**) in mice is associated with protection against colitis, arthritis, allergic responses and pathogen-induced inflammation [11–15]. Administration of this bacterium to humans increases Foxp3+ lymphocytes in peripheral blood, enhances IL-10 secretion *ex vivo*, and reduces the level of circulating proinflammatory biomarkers in a wide range of patient groups and healthy volunteers [16, 17]. A number of host mechanisms have been described, which contribute to the anti-inflammatory activity of this microbe, including TLR-2 and DC-SIGN recognition, and retinoic acid release by dendritic cells [16, 18].

Bifidobacterial strain-specific structures that interact with the host are recently being better described and bifidobacterial-associated exopolysaccharides (EPS) are of particular interest in the field. The genetic content and structure of bifidobacterial EPS clusters has already been described as being highly variable [19]. A number of *in vitro* studies have demonstrated how EPS from members of several bifidobacterial species elicit different imunological responses [20]. For example, an *in vivo* study of EPS produced by *Bifidobacterium breve* UCC2003 showed how it promotes persistence in the gut following colonization through evasion of the adaptive host immune response [21].

In this study we wished to identify and characterize **35624** strain-specific features, such as the **35624** EPS. We sequenced the genome of **35624** and following core genome-based comparisons to related bifidobacterial strains, **35624** was shown to clearly belong to the *B*. *longum* subsp. *longum* phylogenetic group. Furthermore, a **35624**-specific gene cluster, designated *eps*_*624*_, predicted to be responsible for the production of a exopolysaccharide (EPS_624_), was identified. Following detailed chemical analysis, we identified that EPS_624_ contained a branched hexasaccharide repeating unit with two galactoses, two glucoses, galacturonic acid and the unusual sugar 6-deoxytalose.

## Results

### 35624 genome sequencing and comparison to other sequenced *B*. *longum* genomes

**Fig 1:**
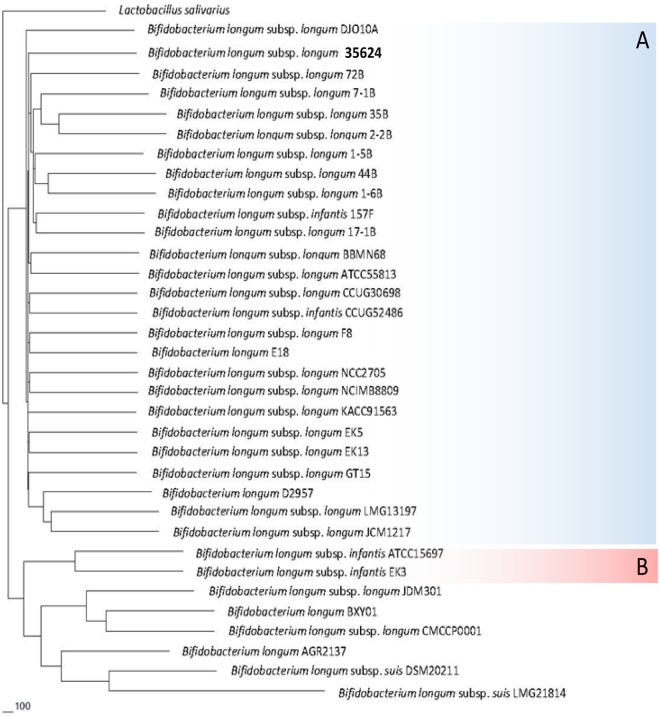
Phylogenetic tree based on the B. longum core-genome. (A) The *B*. *longum* subsp. *longum* phylogenetic group. (B) The *B*. *longum* subsp. *infantis* phylogenetic group. *B*. *longum* subsp. *longum* and *B*. *longum* subsp. *infantis* type strains are indicated in blue text. *Lactobacillus salivarius* was included as an outlier.

The complete genome sequence of the **35624** strain was determined to be 2.26 Mb in size with a G+C % content of 59.34% (for salient features of this genome see Table 1; S1 Fig), consistent with values reported for other bifidobacterial genomes [22]. A total of 1,735 open reading frames (ORFs) were identified, and of these a function could be assigned to 1,370 based on similarity searches to public data bases. In order to accurately evaluate **35624**’s phylogeny and (sub)species assignment, a *B*. *longum* phylogenetic supertree was constructed based on the deduced protein sequences of the *B*. *longum* core-genome [23]. From the resulting supertree, two major clades were identified (Fig 1), of which the largest encompasses twenty six strains of the *B*. *longum* subsp. *longum* phylogenetic group, including the type strain of this subspecies [23]. **35624** had, similar to strains *B*. *longum* 157F and *B*. *longum* CCUG52486, originally been classified as a subspecies *infantis* member. However, classification as based on the phylogenetic supertree approach clearly shows that these three strains are positioned within the *B*. *longum* subsp. *longum* phylogenetic group, and therefore represent *bona fide* members of the subspecies *longum*, their original miss-assignment being due to the sole use of (partial) 16S rRNA-based taxonomic classification [23].

**Table 1:** *Bifidobacterium longum* general genome features.

	*B*. *longum* subsp. *longum* NCIMB 8809	*B*. *longum* subsp. *longum* CCUG 30698	*B*. *longum* 35624	*B*. *longum* subsp. *longum* NCC2705	*B*. *longum* subsp. *longum* DJO10A
Isolated from	Nursling stool	Human adult intestine	Human adult intestine	Infant faeces	Young adult faeces
Genome Size	2.34	2.45	2.26	2.25	2.37
G+C content %	60.1	60.22	59.34	60.12	60.15
Number of identified genes	1872	1983	1735	1727	1990
Percentage of genes functionally assigned	77%	74%	79%	79%	76%
Prophage	1 (complete)	1	0	1	1
Episome	1	0	1	1	1
rRNA copies	3	2	4	4	4
tRNA copies	56	70	56	57	58
CRISPR	0	0	1	1	1

**Fig 2:**
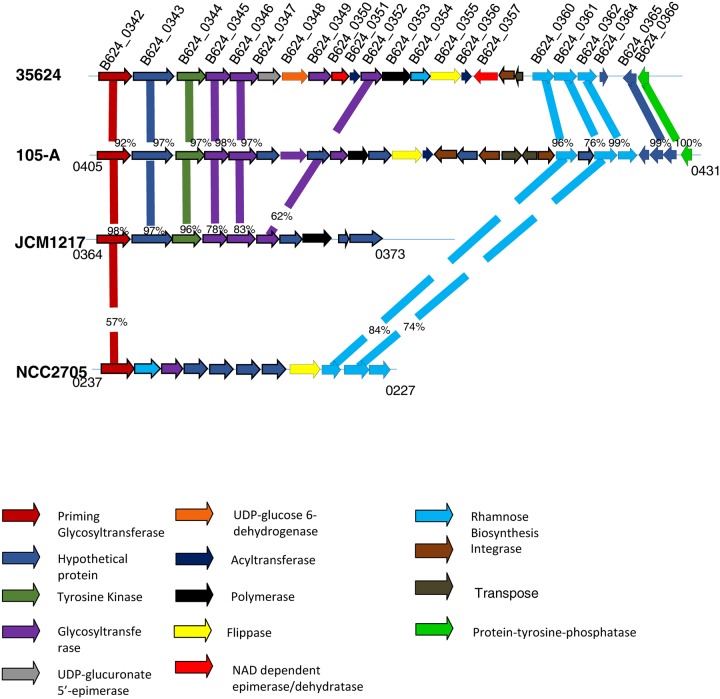
EPS gene cluster. Illustration of the EPS cluster located in the *B*. *longum* **35624** genome and comparison to similar clusters located in *B*. *longum* 105-A, *B*. *longum* subsp. *longum* JCM1217 and *B*. *longum* subsp. *longum* NCC2705. Each gene is colour-coded according to function which is indicated in the legend located at the end of the page. Percentages represent the percent of sequence similarity at the protein level with corresponding genes in the *B*. *longum* **35624** genome. The locus tags of the first and last genes located in the EPS clusters of *B*. *longum* 105-A, *B*. *longum* subsp. *longum* JCM1217 and *B*. *longum* subsp. *longum* NCC2705 are also indicated in the illustration.

Exploration of the **35624** genome revealed the presence of homologs of the *tad* locus which is known to be responsible for the biosynthesis of Type IVb pili, required for host colonisation [24]. Interestingly, **35624** is not predicted to encode so-called sortase-dependent pili, which have previously been shown to play a role in host-microbe interactions [25]. Comparative analysis was performed between the **35624** genome and other *B*. *longum* genome sequences in the hope to locate genetic elements specifying other extracellular structures that may be involved in such interactions and that would exhibit 35624-specific attributes. The main genomic difference between **35624** and other *B*. *longum* genomes is the presence of a 26.2 Kb gene cluster, designated here as *eps*_*624*_ (Fig 2), predicted to encode the biosynthetic machinery for EPS biosynthesis. Comparative analysis of complete and publicly available *B*. *longum* subsp. *longum* genomes shows that a predicted EPS-specifying gene cluster is present in the same location in other *B*. *longum* subs. *longum* genomes, but that the genetic composition of these gene clusters is very diverse at intraspecies level and that the *eps*_*624*_ gene cluster only displays partial similarity to three other EPS-specifying gene clusters (Fig 2). This analysis furthermore reveals that some of these EPS gene clusters lack certain critical functions required for EPS synthesis (Fig 2). For example, the EPS cluster in *B*. *longum* subsp. *longum* JCM1217 appears to lack a flippase-encoding gene which is responsible for the export of EPS precursors across the cell membrane. These findings are in agreement with a recent study [23].

Notably, the *eps*_*624*_ cluster encodes a number of key enzymes that are predicted to be required for EPS production by means of the so-called Wzx/Wzy-dependent pathway, which typically employs a priming glycosyltransferase (pGT), one or more glycosyl transferases (GHs), a flippase, and a polymerase to produce an extracellular heteropolysaccharide [19, 26]. The first gene (corresponding to locus tag BL_0342 and designated here as *pgt*_*624*_) of the *eps*_*624*_ gene cluster is predicted to encode the pGT, which adds the first monosaccharide to a cytoplasmic, membrane-bound carrier molecule undecaprenyl as part of the oligosaccharide subunit biosynthesis [27]. The *eps*_*624*_ cluster encodes five additional GTs (corresponding to locus tags BL_0345, BL_0346, BL_0349 and BL_0352; Fig 2), which are predicted to each add one monosaccharide to the carrier molecule so as to complete the oligosaccharide subunit, prior to its export to the external side of the membrane by a flippase (predicted to be encoded by a gene corresponding to locus tag BL_0355) and its subsequent use by a polymerase (putatively specified by locus tag BL_0353) to produce the EPS polymer. Interestingly, two adjacent genes of the *eps*_*624*_ cluster, corresponding to B624_0347 and B624_0348, are predicted to encode a UDP-glucuronate 5'-epimerase and a UDP-glucose 6-dehydrogenase, suggesting that one of the incorporated monosaccharides of the EPS is an epimer of glucuronic acid, e.g. galacturonic acid or mannuronic acid. Three genes located within the *eps*_*624*_ cluster, corresponding to locus tags B624_0360 through to B624_0362 (Fig 2), encode enzymes known to be involved in the biosynthesis of dTDP-L-rhamnose [19, 28, 29], while the deduced protein products of B624_0350 and B624_0357 are predicted to encode NAD-dependent reductase/epimerase enzymes. Such enzymes have been shown to be involved in the rerouting of the dTDP-L-rhamnose biosynthesis pathway towards the production of dTDP-D-fucose or dTDP-6-deoxy-L-talose [30–32]. The *eps*_*624*_ cluster also encodes two predicted acetyl transferases, similar to enzymes that have previously been shown to perform O-acetylation reactions on particular sugar components (such as 6-deoxy-L-talose) in polysaccharides [33]. Furthermore, the genes with locus tags B624_0344 and B624_0366 represent putative tyrosine kinase and phosphotyrosine protein phosphatase activities, respectively, which have been associated with controlling EPS export, polymerisation and production [34, 35]. Therefore, based on the gene content of the *eps*_*624*_ cluster, we predict that the EPS produced by the **35624** strain is composed of a repeating subunit that consists of six monosaccharides, of which one is an epimer of glucuronic acid, one or two others are either D-fucose or 6-deoxy-talose, and some of which may be O-acetylated. We show below that this prediction is quite correct.

### 35624 EPS isolation

**Fig 3:**
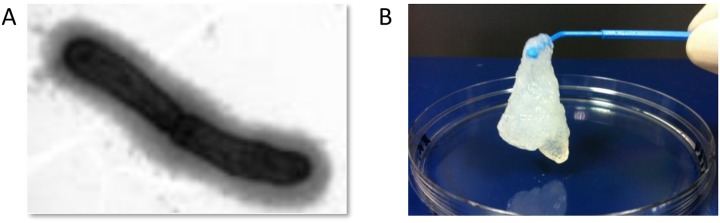
B. longum 35624 electron microscopy. (A) A layer of extracellular polysaccharide is clearly visible by electronic microscopy of the **35624** strain. (B) The isolated and purified EPS is illustrated.

We performed electron microscopy analysis, which indeed revealed the presence of a thick EPS layer on the cell surface of the **35624** strain (Fig 3A). Following harvesting of **35624** cells, which were grown on agar plates to minimize carryover of media components, an EPS solution was generated by agitating the cells in PBS. The harvested EPS solution was mixed with ethanol and the EPS aggregated at the center of the surface of the ethanol solution, which facilitated harvesting of the EPS without the need for centrifugation. The EPS aggregations were taken with a spatula, resuspended in water and dialysed against water to remove contaminants and residual ethanol. Further purification using reverse phase columns resulted in a highly purified polysaccharide, with no detectable proteins or lipids remaining. The precipitated and purified EPS is illustrated in Fig 3B.

### 35624 EPS chemical characterization

**Fig 4:**
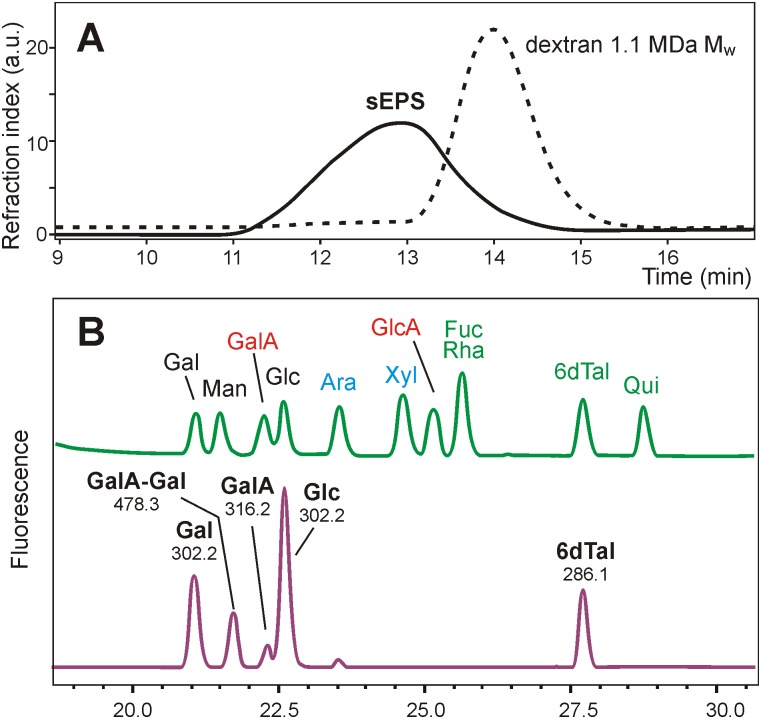
B. longum 35624 EPS characterization. (A) Comparison of the EPS (solid line) with a dextran standards dotted line) demonstrated that EPS had an average molecular mass much higher than 1 MDa (Mw). (B) HPLC analysis of anthranilic acid-labeled monosaccharides of EPS revealed the presence of glucose (Glc), galactose (Gal), some galacturonic acid (GalA) and two additional peaks with masses corresponding to an aldobiuronic acid and a deoxy-hexose later identified as 6-deoxy-talose. The upper trace in (B) is the standard mixture and the lower trace shows the results of the EPS sample. Numbers in the lower trace give the masses of the compounds as determined by off-line ESI-MS.

Comparison of the purified **35624** EPS with dextran standards by high performance size exclusion chromatography (SEC) indicated an average mass in excess of 1 MDa (Mw) (Fig 4A). Monosaccharide analysis of SEC fractions gave the same composition for all fractions of the broad peak. Anion exchange of the EPS revealed it as being negatively charged. ^1^H NMR data recorded at 90°C in D_2_O showed severe line broadening (data not shown) but nevertheless indicated the presence of methyl groups corresponding to acetyl and 6-deoxy protons in an approximate 1:1 ratio.

Analysis of the EPS hydrolysates was accomplished by high performance liquid chromatography (HPLC) of sugars derivatized with either 1-phenyl-3-methyl-5-pyrazolone (PMP) or anthranilic acid using at first the standard solvent systems. This implied the occurrence of glucose (Glc), galactose (Gal) and a small amount of galacturonic acid (GalA) besides two peaks not coeluting with any standard (Fig 4B). Anthranilic acid derivatives were then analyzed with a volatile buffer allowing mass spectrometry of isolated peaks, which revealed one unknown peak as being a disaccharide consisting of uronic acid and a hexose and the other one as a deoxy-hexose that neither was fucose, rhamnose or quinovose (6-deoxyglucose). Gas chromatography-mass spectrometry (GC-MS) of partially methylated alditol acetates identified it as 6-deoxy-hexose (data not shown). With NMR data pointing at 6-deoxy-talose (6dTal), this rare sugar was synthetized from L-fucose by epimerization at C-2 and deoxy-hexose indeed co-eluted with 6dTal (Fig 4B). Adding the peak area of the aldobiuronic acid to that of Gal and GalA, the molar ratio of the constituent sugars Glc, Gal, GalA and a 6dTal approximated 2: 2: 1: 1 respectively.

**Fig 5:**
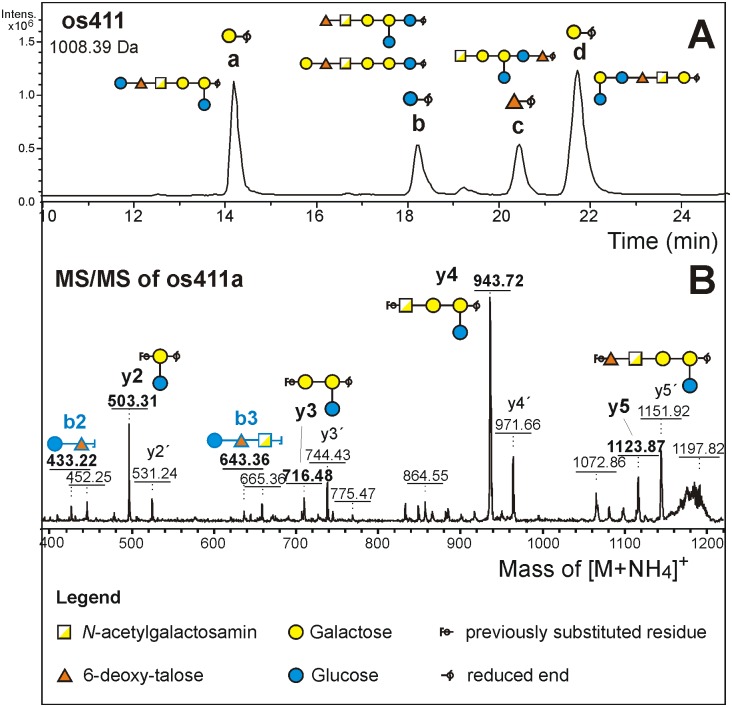
Mild acid hydrolysis of EPS. (A) Separation of EPS fragments by PGC HPLC with MS/MS detection. The extracted ion chromatogram for mass 1008.39 Da shows four peaks. Their reducing end sugar was clearly revealed by ESI-MS/MS. Their assignment as either Gal or Glc and the interpretation in terms of fragment structures was done *a posteriori* based on MALDI-TOF data and on knowledge of the EPS structure. (B) Example of a MALDI-TOF/TOF fragment spectrum showing b-ions from the non-reducing and y- and y´ (= ^1,5^x) -ions from the reducing end.

The products of mild acid treatment were analysed by porous graphitic-liquid chromatography-electrospray ionization-mass spectrometry (PGC-LC-ESI-MS). The occurence of several isobaric fragments indicated lack of a preferred cleavage site. The smallest of the major fragments was the tetrasaccharide os211 consisting of 2 Hex, 1 GalA and 1 dHex residue. ESI-MS/MS of the BD_4_-reduced oligosaccharide identified its reducing end as hexose. Os211 was present as a single isomer, which allowed the preparative isolation of os211 by hydrophilic interaction HPLC for NMR analysis. The os311 and os411 fragments, when analyzed by PGC-LC-ESI-MS/MS turned out to contain at least 3 isomers with hexose and 1 with deoxyhexose at the reducing end (Fig 5A). Reducing end dHex on os411 and larger fragments implies this sugar to be part of the main chain rather than a side arm. These isomers were isolated by preparative PGC for MS/MS in permethylated form.

### 35624 EPS linkage analysis

Permethylation of the intact polysaccharide failed due its insolubility in DMSO. Results were obtained upon gentle hydrolysis with 50 mM TFA (4 h 80°C). Peaks for a 4-substituted 6-deoxy-hexopyranose (or a 5-substituted furanose), terminal Glc, 4-substituted Gal and 2,4-disubstituted Gal were obtained in a ratio of approximately 0.8: 1: 0.6: 1: 1. A uronic acid cannot be seen by this approach. Taking into account the incomplete hydrolysis of the uronic acid´s glycosidic linkage as its acid function is regained during the hydrolysis step and the higher volatility of dHex, this insinuates that each linkage variants occurs just once in the repeating unit. The low yield of 4-substituted Gal pointed at its substitution by uronic acid, which results in a particularily acid stable linkage.

Permethylation linkage analysis of fragment os211 revealed terminal Glc, 4-substituted 6-deoxy-hexose and a 4-substituted Gal at the reducing end. The occurrence of two singly substituted and one terminal residue necessitates a linear topology of os211. As the occurrence of aldobiuronic acid implies a HexA-Hex sequence, the only topology of os211 consistent with the hitherto findings is Glc-d6Tal-GalA-Gal.

The chromatographically separated, reduced and perdeuteromethylated EPS fragments were analyzed by MS/MS on a MALDI-TOF/TOF instrument (Fig 5B). The single os211 fragment gave one y and one b fragment, that underpinned the Glc-dHex-GalA-Gal topology. In the spectra of os311a and os411a, y-fragments with two and three hexoses and with three hexoses plus uronic acid were found. Necessarily, these y-fragments must have harbored the branching Gal residue bearing the unsubstituted Glc residue. The topology GalA-(Glc-)Gal-Gal, however, could not lead to a fragment of m/z 503.3, with only one fragmentation char. Rather, the MS/MS spectra for os311a and os411a could be reconciled with the fragment structure GalA-Gal-(Glc-)Gal. Os311a did not show a b-fragment from the non-reducing terminus but os411a presented the disaccharide Hex-dHex (= Glc-dTal). Thus, together with the insights from linkage analysis of os211, os311a could be assigned the sequence d6Tal-GalA-Gal-(Glc-)Gal and os411a Glc-d6Tal-GalA-Gal-(Glc-)Gal, which is the hexasaccharide proposed to constitute the repeating unit of **35624** EPS and which would also be fully consistent with the genetic content of the *eps624* cluster (see above).

**Fig 6:**
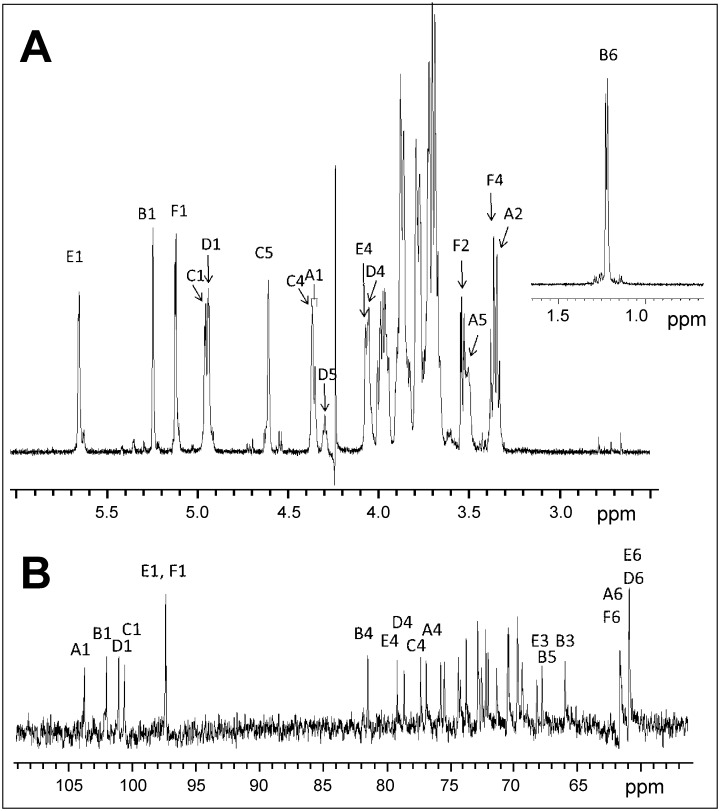
B. longum 35624 EPS characterization. (A) The 600 MHz ^1^H NMR proton spectrum of the acid-treated **35624** EPS (D_2_O, 338 K) is illustrated. A part of the high-field region is displayed in the insert. **(B)** Expansion plot of the 150 MHz 13C NMR spectrum of the acid-treated **35624** exopolysaccharide. The anomeric signals on the left confirmed the presence of a hexasaccharide repeat unit.

The 600 MHz ^1^H-NMR spectra of very mild (as for permethylation) acid-treated exopolysaccharide sample were recorded in D_2_O at 297 K and 338 K, respectively. Since the latter condition led to better resolved signals, the ensuing ^13^C NMR as well as COSY, TOCSY, HSQC-TOCSY, HMBC and ROESY spectra were recorded at 338 K throughout. The proton spectrum (Fig 6A) revealed inter alia six signals of equal intensity attributable to anomeric protons, which gave HSQC-correlations to connected carbons in the range of 97–104 ppm. The absence of anomeric carbon signals shifted to lower-field than 104 ppm and of any signals of non-anomeric carbons at a field lower than 82 ppm confirmed that furanose forms were not present [36]. Five of the anomeric signals (**B1-F1**) had small homonuclear nuclear coupling constants, while residue A displayed a larger coupling constant J_1,2_ (~ 8 Hz). The assignment of the α-anomeric configuration for pyranose residues **B-F** was confirmed by the values of the heteronuclear coupling constant J_C-1,H-1_ (observed in an HMBC experiment) that were found in a range of 170–176 Hz. The coupling constant J_C-1,H-1_ for residue **A** was consistent with the β-anomeric configuration (167.9 Hz). In the high-field segment of the ^1^H-NMR spectrum a methyl group signal being characteristic of a 6-deoxy-pyranose was found at 1.22 ppm.

In the low-field section of the spectrum, two additional, non-anomeric and spin-coupled proton signals were seen (**C**4, **C**5). The signal observed at 4.62 ppm (**C**5) revealed an HMBC connectivity to a carbon signal at 175.3 ppm, thus indicating the presence of a pyranosyluronic acid (**C**).

The presence of a hexasaccharide repeat unit was confirmed by analysis of the 150 MHz ^13^C NMR spectrum which contained four signals of anomeric carbons in the region of 100–104 ppm (**A-D**), and a slightly high-field shifted signal of double intensity at 97.5 ppm (**E,F**) (Fig 6B). Pyranose ring carbon signals were present in the range between 65.9 and 81.5 ppm, while methylene carbons (identified via an APT experiment) were shown as two broadened singlets of double intensity at 61.6 and 60.9 ppm, respectively. Additionally, a carbonyl signal originating from the uronic acid moiety was observed at 175.3 ppm, as well as the carbon signal of the methyl group of the 6-deoxy sugar at 16.4 ppm.

**Fig 7:**
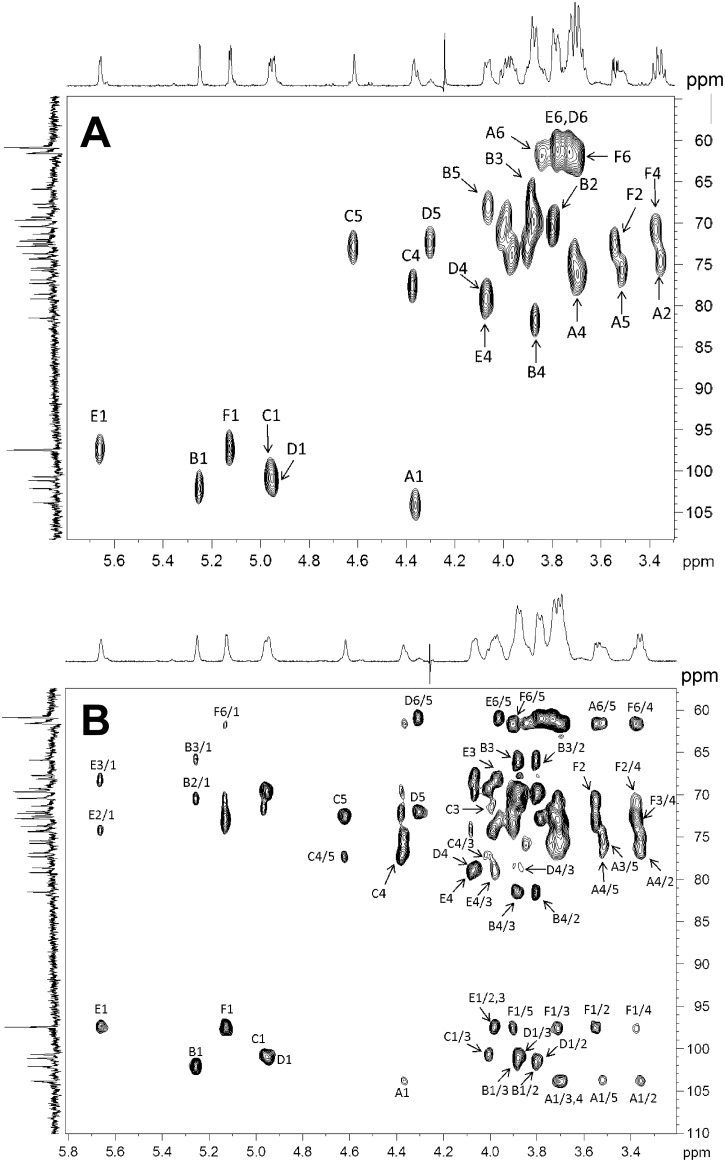
B. longum 35624 EPS proton and carbon signals. (**A**) A selected region of the multiplicity-edited, gradient enhanced ^1^H, ^13^C-HSQC NMR spectrum of the exopolysaccharide. Letters denote the residues as given in the structural formula and arabic numerals denote the respective pyranose position. Resonances from anomeric carbons/protons, glycosylation sites and resolved signals are annotated. (**B**) Selected region of the ^1^H, ^13^C-HSQC-TOCSY NMR spectrum (600 MHz) of the acid-treated **35624** EPS. Arabic numerals before and after oblique stroke denote carbons and protons, respectively.

Proton and carbon signals were then assigned using COSY, TOCSY, HSQC, HSQC-TOCSY (Fig 7A and 7B), HMBC and ROESY experiments (Table 2). Glycosylation shifts were seen for units **A-E**, whereas residue **F** occurred as an unsubstituted sugar. Glycosylation sites were identified at position 4 of residues **A-E**. Residue **E** was found to be additionally substituted at carbon 2, based on an HMBC correlation from H-1 of **F** to C-2 of **E**. Furthermore, both anomeric protons of residues **E** and **F** gave inter-residue ROEs in support of a close spatial proximity. The observed substitution pattern was thus in full agreement with the results of the permethylation analysis.

**Table 2:** ^1^H and ^13^C NMR chemical shifts (δ, ppm) of the exopolysaccharide (recorded at 338 K) and the tetrasaccharide os211 (recorded at 300 K) from *B*. *longum* 35624.

Sugar residue	H-1	H-2	H-3	H-4	H-5	H-6 (6a,6b)
	C-1	C-2	C-3	C-4	C-5	C-6
→4)-β-D-Glcp-(1→	4.35	3.36	3.68	3.70	3.51	3.70, 3.84
A	103.83	74.36a	75.46b	76.90	75.76b	61.62
→4)-α-L-6-deoxy-Talp-(1→	5.25	3.80	3.88	3.86	4.06	1.22
B	102.10	70.49c	65.93	81.50	67.74	16.38
→4)-α-D-GalpA-(1→	4.96	3.87	4.00	4.38	4.62	-
C	100.69	69.66	71.34	77.33	72.84	175.26
→4)-α-D-Galp-(1→	4.94	3.80	3.88	4.06	4.30	3.73
D	101.16	69.31	70.43c	78.63	72.22d	60.89
→2,4)-α-D-Galp-(1→	5.66	3.97	3.98	4.08	3.96	3.78
E	97.47	74.22a	68.16	79.19	72.02d	60.89
α-D-Glcp-(1→	5.13	3.54	3.71	3.38	3.90	3.71
F	97.47	72.54	73.74	70.80	72.80	61.62
Tetrasaccharide os211	H-1	H-2	H-3	H-4	H-5	H-6 (6a,6b)
	J (Hz)	J (Hz)	J (Hz)	J (Hz)	J (Hz)	J (Hz)
→4)-β-D-Glcp-(1→	4.385	3.35	3.46	3.39	3.40	3.71, ~3.86
A	7.9	8.8	9.0			4.9, 12.5
→4)-α-L-6-deoxy Talp-(1→	5.275	3.84	3.915	n.d.	4.08	1.25
B	2.3		3.3		2.4	6.6
→4)-α-D-GalpA-(1→hexitol)	5.07	3.90	4.04	4.38	4.32	-
C	3.8	10.2	2.5	1.9	n.d.	

^a,b,c,d^ assignments may be reversed

The absolute configuration of constituent sugars was derived from the analysis of the trimethylsilylated L-cysteine methyl ester [37] and the results of the monosaccharide composition analysis, this led to the assignment of a D-*gluco* configuration for residues **A** and **F** and a D-*galacto* configuration for residues **C**, **D** and **E**, respectively.

Key assignments were then obtained and confirmed from the tetrasaccharide fragment os211 generated by acid treatment (0.25 M TFA, for 3 h at 80°C) of the EPS followed by borohydride reduction and chromatographic purification. While data of the reduced hexitol component could not be extracted due to significant signal overlap, the proton signals of three pyranose units **A**, **B**, **C** could be fully assigned based on COSY and TOCSY experiments. This allowed to identify the configuration of the constituent sugars based on homonuclear *J*_H,H_ coupling constants as well as ^1^H chemical shift information (Table 2). Residue **A** was assigned as a β-glucopyranosyl residue as seen from the *J*_2,3_ and *J*_3,4_ coupling constants being consistent with a *trans*-diaxial arrangement of the respective protons. Signals arising from residues **B** and **C** were close to those observed for the related units in the polysaccharide chain, whereas H-3 and H-4 signals of unit **A** were shifted to higher field, indicating that hydrolysis had occurred at the residue preceding unit **A**. The small values of the coupling constants *J*_1,2_, *J*_3,4_ and *J*_4,5_ in conjunction with the large values seen for *J*_2,3_, identified residue **C** as α-galacturonic acid, whereas residue **B** corresponds to an α-anomeric 6-deoxy-sugar. The coupling constants for the low-field shifted H-5 and H-3 protons of residue **B** revealed small values for *J*_4,5_, *J*_3,4_ and *J*_2,3_, respectively, which are compatible with the configuration of a 6-deoxy-talose or a 6-deoxy-gulose.

The absolute configuration of the 6-deoxy-sugar was eventually based on the molar rotation values calculated for the polysaccharide. Published optical rotation values of methyl glycosides were used for the calculation [38]. For a hexasaccharide repeat unit containing an α- and β-D-glucopyranosyl unit, two α-D-galactopyranosyl units, one α-D-galactopyranosyluronic residue and a 6-deoxy-α-talopyranose unit, the calculated molar rotation [M_D_] gives 1221.5 for 6-deoxy-α-D-talose as constituent sugar and 844.3 for the presence of 6-deoxy-α-L-talose, which would correspond to optical rotation values of +126 and +87, respectively. The measured optical rotation value [α]_D_^20^ +84.7 (*c* 0.96, H_2_O) was in full agreement with the presence of a 6d-L-talopyranose. This assignment was further supported by characteristic chemical shift differences observed for anomeric carbons when engaged in linkages between L,D- and D,D-configured sugars [39, 40]. The presence of rhamnose, fucose and 6-deoxy-quinovose was excluded based on the results of the HPLC monosaccharide analysis, while the ^13^C NMR chemical shifts observed for residue **B** were not compatible with the presence of a 6-deoxy-gulose, 6-deoxy-altrose and 6-deoxy-allose [39]. The NMR data of the 6-deoxy-pyranose showed a diagnostic high-field shifted ^13^C NMR signal as seen for C-3 of talose^1^ and were also in good agreement with published NMR features of a disaccharide fragment β-D-Glc*p*-(1→4)-α-L-6dTalp occurring in *Burkholderia caribensis* strain MWAP71 [41]. The identity of the deoxy-sugar was eventually proven using a synthetic sample of L-6dTal.

HMBC data (two experiments with values of *J*_CH_-coupling constants of 5 and 11 Hz, respectively, were performed) confirmed the assignment of spin systems with characteristic intraresidue correlations between the anomeric protons of residues **C** and **F** to carbon 3, and anomeric protons of residues **A**, **B** and **E** to carbon 5, respectively. In addition the following inter-residue connections were observed: H-1 of **A** and C-4 of **B**, H-1 of **B** and C-4 of **C**, H-4 of **E** and C-1 of **D**, H-1 of **F** and C-2 of **E**.

**Fig 8:**
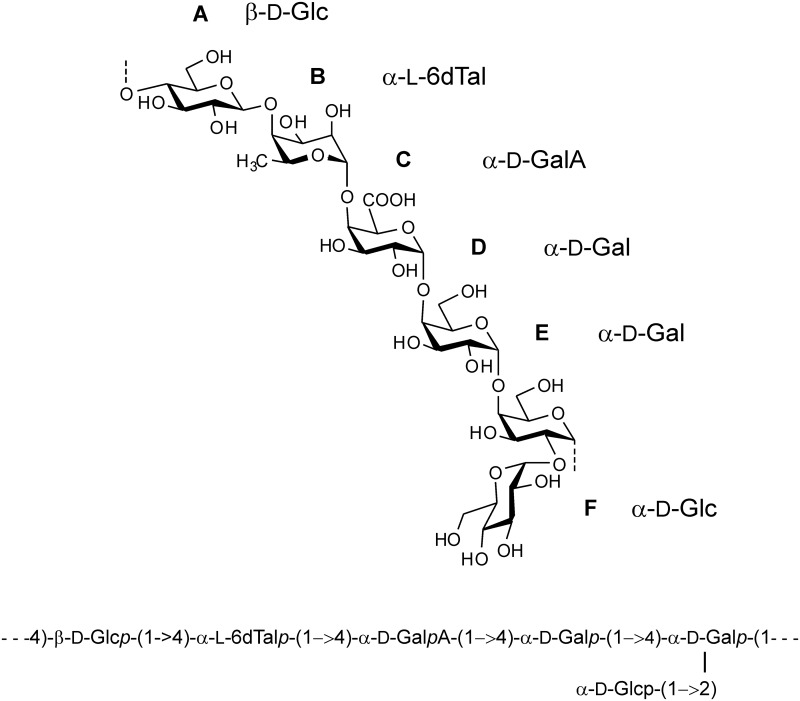
B. longum 35624 EPS composition and structure. The structure is annotated as the chemical formula and in condensed form. Capital letters denote the residues as in Figs 6 and 7.

Based on the combined evidence of sugar analysis, methylation data and NMR-data, the **35624** EPS structure is illustrated in Fig 8.

## Discussion

Bifidobacteria comprise a significant proportion of the gut microbiota, in particular in infants, and many strains are used as probiotics. Strain-specific beneficial effects are well established and the genetic or structural differences that associate with such bifidobacterial strains are beginning to emerge. In this report, we describe a **35624**-specific gene cluster, responsible for the production of a cell surface-associated exopolysaccharide (EPS). The **35624** EPS consists of a branched hexasaccharide repeating unit with two galactoses, two glucoses, galacturonic acid and the infrequent sugar 6-deoxytalose.

Bifidobacterial cell surface-associated polysaccharides have previously been proposed to (i) mediate some of their health benefits, (ii) to aid in their tolerance to the harsh conditions within the gut, and (iii) to influence composition of the gut microbiome by being used as a growth substrate by other microbes [21, 27, 42, 43]. In general, bacterial EPS consists of repeating mono- or oligosaccharide subunits connected by varying glycosidic linkages, which are structurally diverse. As previously reported, and as indicated in our genome comparisons, the identified genes associated with the EPS biosynthetic machinery are highly diverse among the analyzed bifidobacteria, likely contributing to strain-specific traits due to the expected structural and therefore functional diversity of such EPS molecules [19]. Of note, pathogen-associated EPSs have long been known to be critical in host–microbe interactions, where they facilitate adherence and colonization within the human host, with additional immunomodulatory effects [19, 44, 45]. Regarding the role of EPS in counteracting pathogens, a role of scavenger and trapping system to recruit and present pathogens to host cell receptors has been proposed [46]. With respect to gastrointestinal infections, the EPS produced by *B*. *longum* BCRC 1464 has been shown to possess antimicrobial activity against pathogens and immune-modulating activity, which causes release of the anti-inflammatory cytokine IL-10 [47]. Furthermore, EPS of *B*. *animalis* subsp. *lactis* has been shown to act as an adherent surface trapping pathogens on the mucus layer [46] and exopolysaccharide capsule production by a *B*. *breve* strain was also shown to be linked to the evasion of adaptive B-cell responses [21].

Previously, the **35624** strain has been described as *B*. *longum* subsp. *infantis* according to the most up to date information available at that time for classifying bifidobacteria subspecies. However, following the full sequencing of the bacterial genome and the use of its core genome for phylogenetic taxon assignment, it is now apparent that this bacterium should be reclassified as *B*. *longum* subsp. *longum*. Clearly, the reclassification of the strain does not affect the strain-specific beneficial effects previously described for **35624**. However, very few probiotic strains that are commercially available have been characterized to the same extent in terms of mechanism and genome identification as the **35624** strain and it is likely that without such in-depth analysis many probiotic products have been incorrectly classified and labeled. Lack of clarity on the key mechanism of action associated with other probiotic products is compounded by the fact that many contain multiple strains with unknown interactions.

Polysaccharides from *B*. *bifidum*, *B*. *breve*, *B*. *infantis* and *B*. *longum* strains typically contain glucose and galactose, with rhamnose occasionally being described [48, 49]. The presence of 6-deoxy-L-talose in bifidobacterial polysaccharides has only been previously observed once in a *B*. *adolescentis* strain [50]. However, the repeating unit described herein for the **35624** strain has never been previously described in bifidobacteria. The occurrence of 6-deoxy-L-talose has been previously described in unrelated microbial species, which include *Agrobacterium rubi*, *Rhizobium loti*, *Treponema pectinovorum*, *Burkholderia pseudomallei*, *Mycobacterium intracellulare*, *Mycobacterium smegmatis*, *Aeromonas hydrophila*, *Pseudoalteromonas flavipulchra*, *Hafnia alvei*, *Actinobacillus actinomycetemcomitans*, and *Proteus* strains [51–62]. Many of these organisms are Gram-negative bacteria and the presence of 6-deoxy-talose in their LPS and in the EPS from the Gram-positive *B*. *longum* **35624** suggests that specific monosaccharides can be shared in the surface associated polysaccharides of widely divergent bacteria. In most cases, 6-deoxy-L-talose has been reported to be *O*-acetylated at position 2 and 4, respectively.

In conclusion, we have identified a specific genetic locus associated with the biosynthesis of a EPS in the human commensal *B*. *longum* **35624** strain. This EPS possesses a chemical structure that has not been previously described for other bifidobacterial strains. Future studies will assess if the **35624** EPS has any immunoregulatory effects.

## Materials and Methods

### Bacterial culture conditions

Bifidobacteria were routinely cultured in either de Man Rogosa and Sharpe medium (MRS; Oxoid Ltd., Basingstoke, Hampshire, United Kingdom) supplemented with 0.05% cysteine-HCl or reinforced clostridial medium (RCM; Oxoid Ltd.). Bifidobacterial cultures were incubated at 37°C under anaerobic conditions in a Don Whitley anaerobic Chamber. Modified Man Rogosa and Sharpe agar plates, containing 3% glucose, were used to grow **35624** for EPS extraction and purification.

### Genome Sequencing and data assembly

Chromosomal DNA from bifidobacteria was isolated as previously described [63]. The genome sequence of **35624** was sequenced using a Roche 454 FLX Titanium instrument by the commercial sequencing service providers Agencourt Bioscience (Beverly, MA) and Eurofins MWG Operon (Germany) and then assembled, after which remaining gaps were closed using Sanger Sequencing of PCR products. Sequence reads were initially assembled using Phred [64, 65], Phrap (P. Green, University of Washington; http://www.phrap.org/), RepeatMasker (AFA. Smit, R. Hubley, & P.Green; www.repeatmasker.org/) and the Staden software package [66]. Final assembly of the **35624** genome was verified using Newbler v 2.3 (http://454.com/products/analysis-software/index.asp). The accession number for the **35624** genome sequence is CP013673 (NCBI—http://www.ncbi.nlm.nih.gov/).

### General features prediction

Prediction of putative open reading frames (ORFs) was performed using PRODIGAL prediction software (http://prodigal.ornl.gov/) and supported by BLASTX [67] alignments. Results of Prodigal/BLASTX were combined manually and a preliminary identification of ORFs was performed on the basis of BLASTP analysis against a non-redundant protein database provided by the National Centre for Biotechnology (http://www.ncbi.nlm.nih.gov/). Using the ORF finding outputs and associated BLASTP results, Artemis [68] was employed for visualisation and manual editing in order to verify, and if necessary, redefine the start of every predicted coding region, or to remove or add coding regions. The assignment of protein function to predicted coding regions was performed manually. In addition, the individual members of the revised gene/protein data set were searched against the protein family (Pfam) [69] and COG [70] databases. Ribosomal RNA (rRNA) and transfer RNA (tRNA) genes were detected using RNAMMER (http://www.cbs.dtu.dk/services/RNAmmer/) and tRNA-scanSE (http://lowelab.ucsc.edu/tRNAscan-SE/), respectively.

### Phylogenetic analysis

The computation of a phylogenetic supertree was performed based on the alignment of a set of orthologous proteins defined by the pan-genome computation. Each protein family was aligned using CLUSTAL_W v1.83 [71]. Phylogenetic trees were computed using the maximum-likelihood in PhyML v3.0 [72] and concatenated; the resulting consensus tree was computed using the Consense module from the Phylip package v3.69 using the majority rule method (http://evolution.genetics.washington.edu/phylip.html).

### 35624 EPS isolation

The **35624** strain was cultured on MRS agar plates at 37°C under anaerobic conditions for 60 h. Cells were harvested using cell scrapers and resuspended for 2 h with constant rotary agitation in PBS containing Ribonuclease A (1 μg/ml) and Deoxyribonuclease I (5 μg/ml Sigma, St. Louis, MO, USA). Whole cells were removed by centrifugation for 30 min at 4°C and 20,000 x *g* (Sorval RC6 plus) and the supernatant was filtered through 0.45 μm syringe filters. The EPS-containing solution was poured into 3 volumes of chilled ethanol, gently stirred for 30 seconds and incubated at 4°C for 2 h. The precipitated EPS located at the surface of the ethanol was removed with a spatula and resuspended in H_2_0 under constant, yet gentle shaking for 2 h. The solution was dialysed against H_2_0 in a dialysis tubing (14 kDa, Sigma) at 4°C and consequently applied 2 times on SPE C18 columns (Bakerbond) as indicated by the manufacturer using a HyperSep-96^™^ vacuum manifold (Thermo Scientific). The flow-through fraction was collected and filtered through 0.45μm syringe filters. The solution was aseptically filled in glass vials and freeze-dried (Virtis Genesis). Dried EPS was stored at -80°C until further analysis.

### 35624 EPS characterization and composition

Size exclusion chromatography was performed with a PL aquagel-OH MIXED-H 8 μm column (300 x 7.5 mm, Agilent, Waldbronn, Germany) at flow rate of 0.5 mL/min at room temperature with 25 mM ammonium acetate (pH 8.5) and a refractive index detector. Dextran standards (Sigma) with nominal masses (Mw) of 25, 150 and 1185 kDa were used for comparison.

Anion exchange chromatography was performed with EconoPac-Q cartridge (Bio-Rad, Vienna, Austria). The EPS was applied in 50 mm ammonium acetate and eluted with a NaCl gradient. Fractions were analyzed for carbohydrate by the orcinol-sulfuric acid method.

EPS was hydrolyzed with 4 M trifluoro acetic acid at 100°C for 4 h. The monosaccharides were derivatized with anthranilic acid and analyzed by reversed phase HPLC using a 5 μm Kinetex C18 core-shell column (Phenomenex, Torrens, CA) and an acetonitrile gradient in either 0.2% 1-butylamin, 0.5% phosphoric acid, 1% tetrahydrofuran [73] or in 0.3% formic acid buffered to pH 3.0 with ammonia in order to allow subsequent mass spectrometric verification of peaks [74]. High-performance liquid chromatography of monosaccharides as 1-phenyl-3-methyl-5-pyrazolone (PMP) derivatives was performed on a Hypersil ODS column as described [75]. Gas chromatographic analysis of alditol acetates was performed with a 60 m OPTIMA^®^ 1 MS Accent column with 0.25 mm inner diameter and 0.25 μm film thickness (Macherey-Nagel, Düren, Germany) with mass spectrometric detection on a GC System 7820A with coupled to a MSD5975 (both Agilent, Waldbronn, Germany) [76].

For determination of the D/L configuration of Gal and Glc monosaccharides were reacted with l-cysteine methyl ester [37]. The dried sample was then derivatized with N-methyl-N-trimethylsilyltrifluoroacetamide containing 1% trimethylchlorosilane. The sugars were immediately analyzed on an Optima 1MS Accent analytical column (Macherey-Nagel, Germany, 60 m×0.25 mm i.d., 0.25 μm film thickness, 100% dimethylpolysiloxane stationary phase) and detected with a 7200 GC-QTOFMS system (Agilent, Waldbronn, Germany) [77]. Notably, compounds were chemically ionized using methane to give molecular ions such that peaks arising from hexoses, deoxyhexoses and uronic acids could be discriminated. The dominating ion for hexoses was the penta-trimethylsilyl derivative associated with an m/z of 658.2931.

### 35624 EPS linkage analysis

Linkage analysis by gas chromatography with electron impact-mass spectrometry (GLC-MS) was conducted after hydrolysis of EPS under the mildest conditions that provided solubility in dimethyl sulfoxide, *i*.*e*. 50 mM trifluoroacetic acid at 80°C for 4 h. Permethylation was achieved with sodium hydroxide / methyl iodide followed by hydrolysis, reduction with sodium borodeuteride and acetylation. The resulting partially methylated alditol acetates were separated on a 60 m OPTIMA^®^ 1 MS Accent column with 0.25 mm inner diameter and 0.25 μm film thickness (Macherey-Nagel, Düren, Germany). Retention time standards were obtained by derivatizing glucose or galactose with sub-stoichiometric amounts of methyl iodide, which worked well for tetra- and tri-methyl ethers.

### Partial hydrolysis and fragment analysis

EPS was hydrolyzed with 0.25 M TFA at 80° for various times with 4 h giving the best yield of fragments of suitable size. The samples were purified for analysis with porous graphitic carbon (PGC) cartridges (25 mg, Hypersep Hypercarb, Thermo Scientific, Waltham, MA) equilibritated with 0.3% formic acid buffered to pH 3.0 with ammonia and eluted with 50% acetonitrile in this buffer. The eluate was dried, redissolved and separated by PGC HPLC on a 100 x 0.32 mm column (Hypercarb, Thermo Scientific) with a 30 min gradient from 5 to 20% (apart from sample application at 1% and column cleaning up to 50%) acetonitrile in the above formic acid/formate buffer [78]. Detection of compounds was accomplished by electrospray ionization-mass spectrometry (ESI-MS/MS) on a Bruker Maxis G4 Q-TOF mass spectrometer operated in the data dependent acquisition mode. Selected oligosaccharides obtained by preparative HPLC on a 100 x 3 mm hypercarb column (Thermo Scientific) applying the conditions used with the analytical column. Fractions containing relevant oligosaccharides were pooled, dried and permethylated as described above, however, with perdeutero-methyl iodide to allow discrimination between methyl groups and uronic acids. The permethylated oligosaccharides were analyzed as [M+Na]^+^ ions by matrix assisted-time of flight-mass spectrometry using 2,5-dihydroxybenzoic acid as matrix and an Autoflex MALDI-TOF/TOF-MS (Bruker, Bremen, Germany) in the positive reflectron or in LIFT mode.

Preparative isolation of fraction os211 was done by hydrophilic interaction HPLC on a TSKgel Amide-80 column (Tosoh Bioscience, Griesheim, Germany) as recommended by the supplier.

### Nuclear magnetic resonance (NMR) experiments

NMR spectra of the polysaccharide and tetrasaccharide sample were obtained for solutions in 99.9% D_2_O (0.6 mL) at 338 K on a Bruker Avance III 600 instrument (600.2 MHz for ^1^H, 150.9 MHz for ^13^C) equipped with a BBFO broad-band inverse probe head and z-gradients using standard Bruker NMR software TopSpin 3.0. ^1^H spectra were referenced using DSS as standard (δ = 0); ^13^C spectra were referenced using 1,4-dioxane as external standard (δ = 67.40). In general, sweep widths of 5000–6000 Hz for ^1^H and 32000–36000 Hz for ^13^C were used. ^1^H, ^1^H-COSY experiment were measured using the pulse program cosygpqf. ^1^H, ^13^C-HSQC spectra were obtained using the pulse program hsqcedetgp with 1024 x 64 k data points and 600 scans per t_1_-increment. The *J* value for the HSQC and HMBC experiments was 145 Hz for one-bond couplings. Double-quantum filtered ^1^H,^13^C-HMBC experiments were recorded using the pulse program hmbcgpndqf with 4096 x 64 data points, 520 scans per t_1_-increment, and values for long range *J*_X,H_ coupling of 11 and 5 Hz, respectively. The HSQC-TOCSY experiment was performed using the pulse program hsqcdietgpsisp with 1024 x 128 data points and 260 scans per t_1_-increment. The TOCSY experiment was recorded using the program mlevphpp with 1024 x 256 k data points and a mixing time of 120 ms. ROESY spectra were measured using the pulse program roesyphpp.2, 2048 x 256 data points and a ROESY-spinlock pulse of 0.5 sec. Data were processed using a squared sine-bell function and zero filling one time in the f1 dimension.

6-deoxy-L-talose was synthesized as follows. Ethylthio β-L-fucopyranoside was prepared according to literature and was converted into the 3,4-O-isopropylidene derivative at a yield of 93%. While epimerization using Mitsunobu conditions or triflate-displacement failed, oxidation by 2-iodoxybenzoic acid (IBX) followed by selelective reduction of the intermediate 2-ulose proceeded smoothly and afforded the talo-product. Deprotection via hydrolysis of the thioglycoside and cleavage of the 3,4-O-acetonide afforded the efficient production of 6-deoxy-L-talose [74].

## Supporting Information

S1 Fig Genome map of B. longum 35624. The location of the predicted exopolysaccharide gene cluster is illustrated. Track 1:All open reading frames located on the forward strand. Track 2: All open reading frames located on the reverse strand. Track 3: All open reading frames located on the forward strand and colour coded according to functional assignment. Track 4: All open reading frames located on the reverse strand and colour coded according to functional assignment. Track 5: All identified rRNA (indicated in orange) and tRNAs (indicated in green). Track 6: G + C plot and Track 7: The G + C skew.
(TIF)
