# Supplementary figures and images for: Genome Analysis and Characterisation of the Exopolysaccharide Produced by Bifidobacterium longum subsp. longum 35624™
Source: PLoS One. 2016 Sep 22;11(9):e0162983. doi: 10.1371/journal.pone.0162983 (PMC5033381; doi:10.1371/journal.pone.0162983)

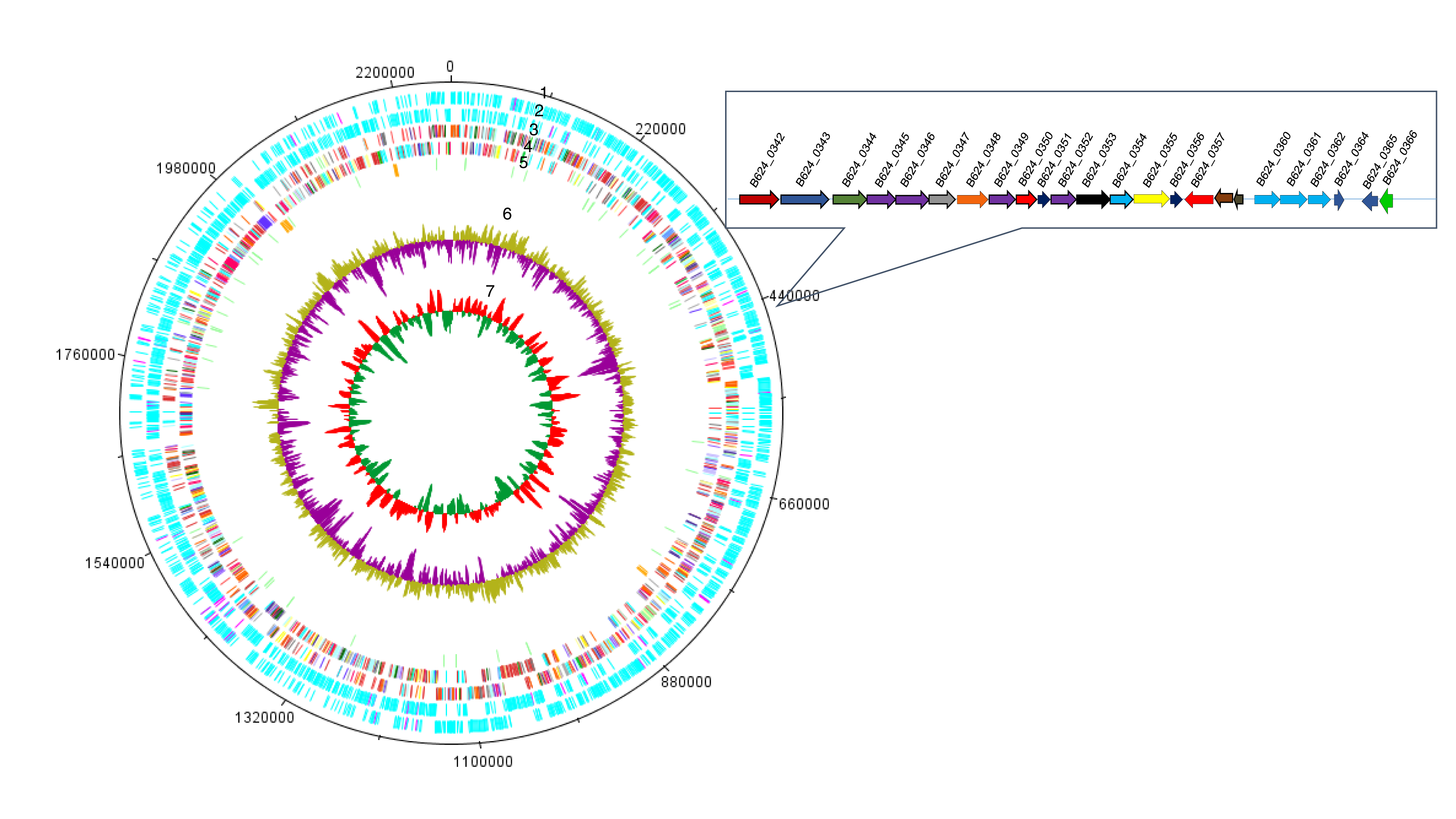

Supplement: S1 Fig — The location of the predicted exopolysaccharide gene cluster is illustrated. Track 1:All open reading frames located on the forward strand. Track 2: All open reading frames located on the reverse strand. Track 3: All open reading frames located on the forward strand and colour coded according to functional assignment. Track 4: All open reading frames located on the reverse strand and colour coded according to functional assignment. Track 5: All identified rRNA (indicated in orange) and tRNAs (indicated in green). Track 6: G + C plot and Track 7: The G + C skew. (TIF) [file pone.0162983.s001.tif]
